# Dynamics of Water Diffusion Changes in Different Tissue Compartments From Acute to Chronic Stroke—A Serial Diffusion Tensor Imaging Study

**DOI:** 10.3389/fneur.2019.00158

**Published:** 2019-02-26

**Authors:** Anna Christina Alegiani, Simon MacLean, Hanna Braass, Susanne Gellißen, Tae-Hee Cho, Laurent Derex, Marc Hermier, Yves Berthezene, Norbert Nighoghossian, Christian Gerloff, Jens Fiehler, Götz Thomalla

**Affiliations:** ^1^Department of Neurology, University Medical Center Hamburg-Eppendorf, Hamburg, Germany; ^2^Department of Neuroradiology, University Medical Center Hamburg-Eppendorf, Hamburg, Germany; ^3^Department of Stroke Medicine, Université Lyon, Lyon, France; ^4^Department of Neuroradiology, Université Lyon, Lyon, France

**Keywords:** acute stroke, stroke, magnetic resonance imaging, diffusion tensor imaging, fractional anisotropy

## Abstract

**Background and Purpose:** The immediate decrease of the apparent diffusion coefficient (ADC) is the main characteristic change of water diffusion in acute ischemic stroke. There is only limited information on the time course of diffusion parameters in different tissue compartments of cerebral ischemia.

**Materials and Methods:** In a longitudinal study, we examined 21 patients with acute ischemic stroke by diffusion tensor imaging within 5 h after symptom onset, 3 h later, 2 days, and 1 month after symptom onset. Acute diffusion lesion and the fluid-attenuated inversion recovery (FLAIR) after 2 days were used as volumes of interest to define persistent core, lesion growth, and reversible acute diffusion lesion. For all diffusion parameters ratios between the stroke lesion VOIs and the mirror VOIs were calculated for each time point. ADC ratio, fractional anisotropy ratios, and eigenvalues ratios were measured in these volumes of interest and in contralateral mirror regions at each time points.

**Results:** In the persistent core, ADC ratio (0.772) and all eigenvalues ratios were reduced on admission up to 1 day after stroke and increased after 1 month (ADC ratio 1.067). Within the region of infarct growth time course of diffusion parameter changes was similar, but delayed. In the brain area with reversible diffusion lesion, a partial normalization of diffusion parameters over the time was observed, while after 1 month diffusion parameters did not show the signature of healthy brain tissue. There were significantly different trends for all parameters over time between the three tissue compartments.

**Conclusion:** Diffusion tensor imaging displays characteristic changes of water diffusion in different tissue compartments over time in acute ischemic stroke. Even regions with reversible diffusion lesion show diffusion signatures of persisting tissue alterations.

## Introduction

The analysis of water diffusion characteristics by diffusion weighted image (DWI) allows for inferences on brain tissue microstructure and integrity (1). Decrease of the apparent diffusion coefficient (ADC) indicates cytotoxic edema occurring within minutes of cerebral ischemia, and as a consequence DWI has become an essential tool in acute stroke imaging (2).

Resulting from tissue organization water diffusion may be different along different directions. Diffusion tensor imaging (DTI) is used to characterize diffusion properties, and fractional anisotropy (FA) is the most commonly used index to characterize the directionality of diffusion. Disintegration of brain tissue and vasogenic edema results in a decrease of FA in chronic stroke (3, 4).

Several distinct compartments of brain tissue can be identified in acute stroke depending on the viable status of tissue, that can be distinguished based on MRI findings and that play an important role in guiding acute stroke treatment. Acute ischemic stroke is a dynamic process. Within the first hours, a growing area of already irreversibly damaged (i.e., infarcted) tissue, usually labeled “ischemic core,” is surrounded by a critically hypoperfused but still viable and potentially salvageable tissue, the “penumbra.” When brain imaging is repeated within the first hours and days of stroke, in most cases a growth of the original ischemic core extending into surrounding penumbral brain tissue can be observed (5).

The brain area with reduced ADC, i.e., the visible DWI lesion, is considered to represent irreversibly damaged tissue. However, normalization of decreased ADC values has been described in both experimental studies and in human stroke, and this observation has challenged the assumption of acute DWI lesions representing irreversibly damaged brain tissue (6). Thus, at least parts of the initial DWI lesion are assumed to represent penumbral tissue.

Altogether, there is only scarce and partly contradictory data on the time course of diffusion changes in different tissue compartments in acute ischemic stroke and questions remain open as to the usefulness of the available diffusion parameters for clinical decision making (7–12). Better understanding of the time course of alterations of the different diffusion parameters might improve the pathophysiological understanding of brain tissue changes in the different relevant tissue compartments and might also add to the definition of irreversibly damaged brain tissue in contrast to potentially salvageable tissue at risk of infarction. The aim of this study was to characterize the time course of water diffusion changes in different compartments of cerebral ischemia from the very first hours of stroke to the chronic stage.

## Materials and Methods

We analyzed data from a subgroup of patients with first-ever anterior circulation stroke from a prospective European multicenter study (I-KNOW) (13). Patients were enrolled at two hospitals (Hospices Civils de Lyon and University Medical Center Hamburg-Eppendorf) between October 2008 and September 2009. Inclusion criteria comprised National Institutes of Health Stroke Scale ≥4 and onset-to-scan time ≤12 h, serial MRI examination including DTI completed at four defined time points (see below). The study was approved by the Ethics Committee of the Hamburg Chamber of Physicians (No. 2666).

### MRI Protocol

Magnetic resonance images were acquired on 1.5 clinical whole-body units (Magnetom Symphony/Sonata; Siemens, Erlangen, Germany). The protocol included a single shot spin-echo echo-planar DTI sequence with diffusion weighting along 12 directions, field of view = 24 × 24 cm, matrix 128 × 128, slice thickness 3 mm, b-value 1,000 s/mm^2^, TR >5,000 s. The protocol also included a conventional single shot spin-echo echo-planar DWI sequence with a TR/TE of 2600/77, slice thickness of 5 mm, 1.5 mm gap, applying three *b* values (0, 500, and 1,000 s/mm^2^). MRI examinations were performed at four time points: T1—on admission (≤12 h of symptom onset); T2—about 3 h after first MRI; T3—about two days after admission; and T4—about 1 month after admission.

### Postprocessing

MRI data were analyzed using FSL (FMRIB Software Library, Center for Functional MRI of the Brain, University of Oxford). The diffusion tensor (D) for each voxel was calculated and maps of FA, mean diffusivity (MD) = apparent diffusion coefficient (ADC), and the three eigenvalues representing diffusivity along the three axes of the diffusion tensor (ʎ1, ʎ2, ʎ3) were calculated. All images were registered to MNI space using linear registration with FSL flirt. CSF was automatically excluded from the segmented brain tissue on diffusion weighted (b = 1,000) images using an upper threshold of 1,200 × 10-6 mm^2^/s (14).

### Volume of Interest Analysis—Acute DWI Lesion, Final Infarct Lesion, Mirror Lesions

**Figure 1:**
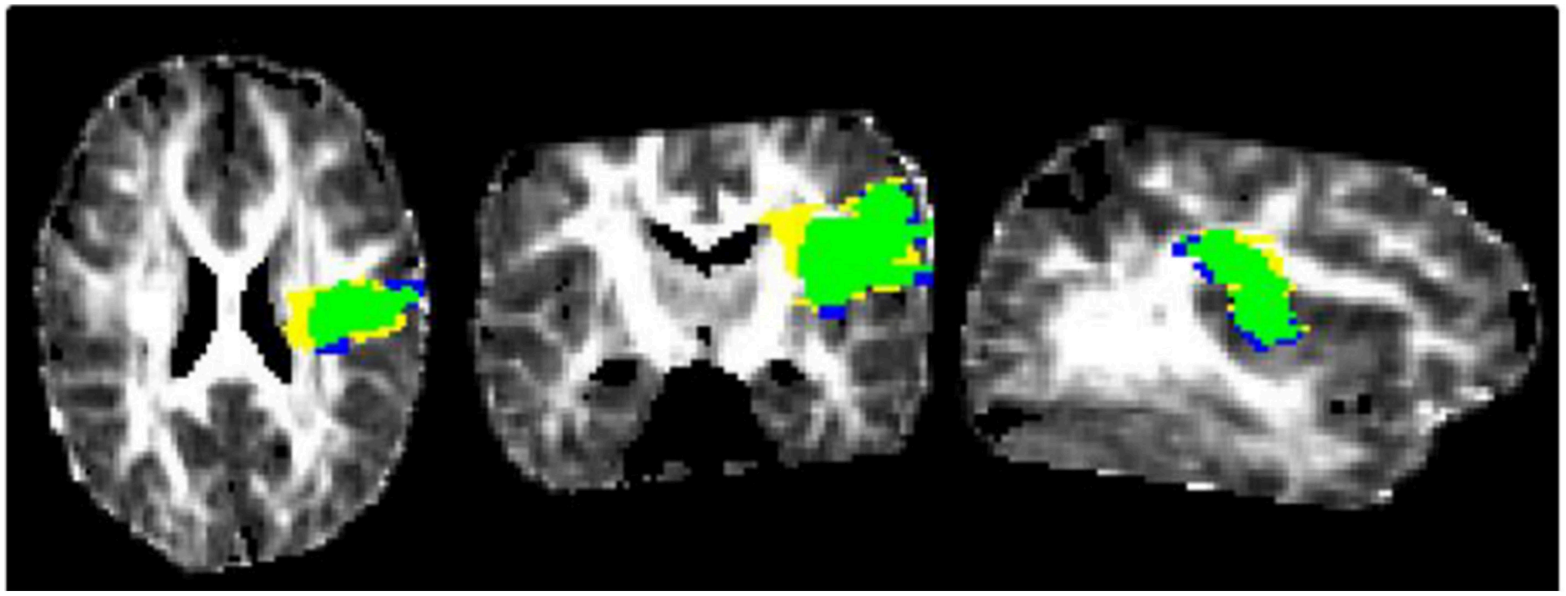
FA at T1, transversal, frontal, and sagittal with persistent core (green) and reversible acute diffusion lesion (yellow) and lesion growth (blue).

Volumes of interest (VOI) of the acute DWI lesion were manually defined on a voxel-by-voxel basis based on visual inspection of ADC maps with Create Mask (FSLview). The final infarct VOIs were defined on the FLAIR-image at T3. We used MRI at T3 to define final infarct lesion volumes in order to avoid problems by strong changes in signal characteristics and possible volume changes resulting from atrophy at a later time point. Minimal VOI size was four voxels on more than one slice (see Figure 1).

### Volume of Interest Analysis—Lesion Masks: Core, Reversal, Growth, Mirror Lesions

**Figure 2:**
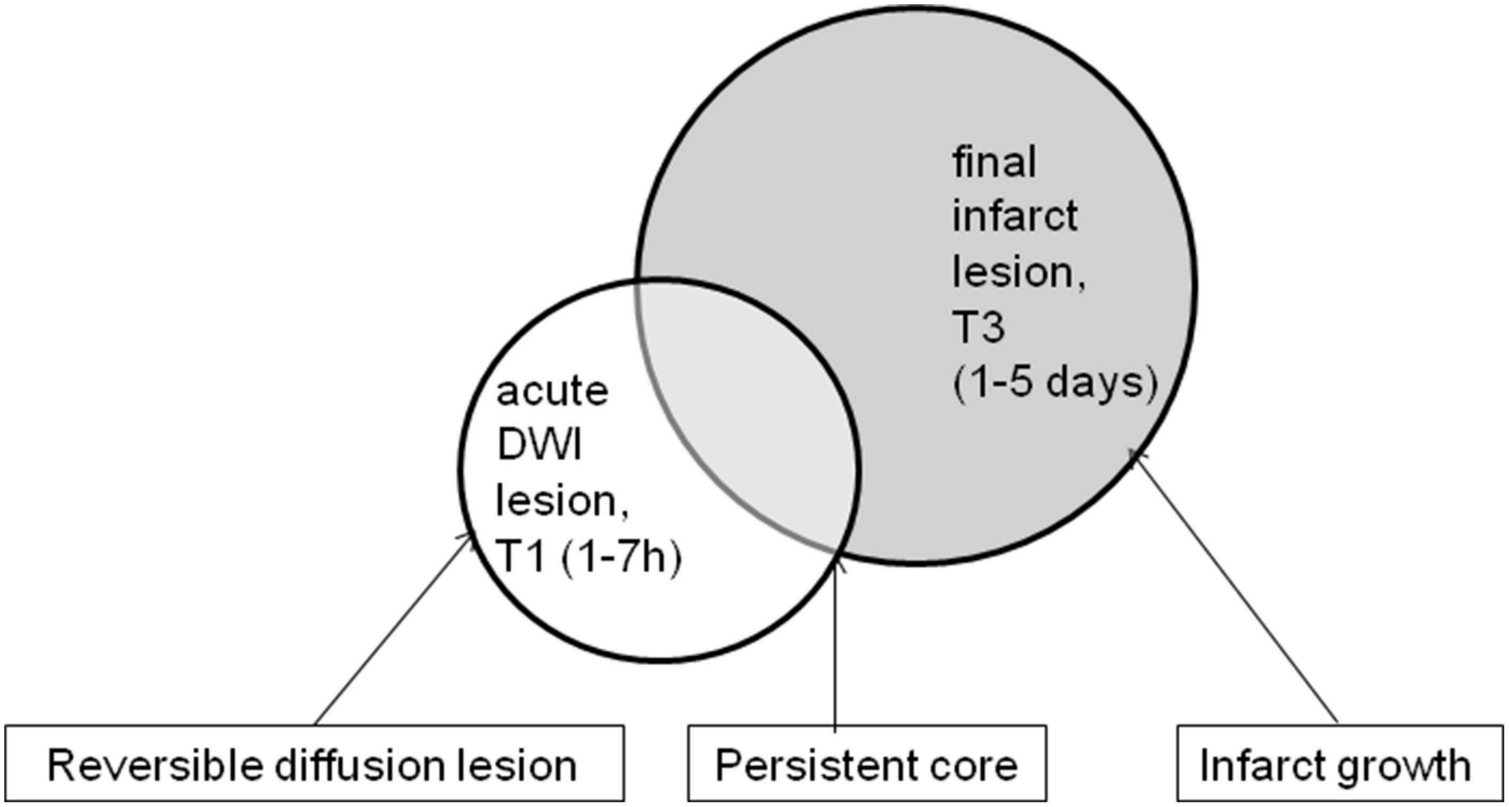
This figure shows the definition of the three different masks respectively tissue compartments: reversible diffusion lesion vs. persistent core vs. infarct growth.

We defined the following three lesion masks representing tissue compartments of interest: persistent core (“core”) = acute DWI lesion ∩ final infarct lesion; infarct growth (“growth”) = final infarct lesion—acute DWI lesion; acute DWI lesion not proceeding to infarction, reversible diffusion lesion (“rev”) = acute DWI lesion—final infarct lesion (Figure 2). All VOIs were mirrored along the x-axis in order to obtain mirror VOIs from the unaffected hemisphere for comparison. Lesion masks were transferred to diffusion parameter maps at all four time points using parameters from linear registration. Values for each VOI were obtained by averaging all voxels within the VOI. For all diffusion parameters (FA and eigenvalues) ratios between the stroke lesion VOIs and the mirror VOIs were calculated for each time point.

### Statistical Analysis

We used SPSS (9.0.1., SPSS Inc., Chicago, IL) for descriptive analysis. All values are presented as mean ± standard deviation or median and interquartile range. The influence of the time course and tissue compartment on the different DTI parameters, were calculated by a mixed model with random intercept (dependent variable: ratio between affected and unaffected side of FA, ADC and eigenvalues; factors: tissue compartment, time-point of imaging). In addition, analysis of FA over time was done by repeated measure ANOVA with a Greenhouse-Geisser correction for each compartment.

## Results

Twenty-one patients met the inclusion criteria and were included in the analysis, 50% were female, median age was 65 years. Median NIHSS on admission was 9. Mean time from symptom onset to first MRI was 3 h 9 min, mean time from symptom onset to the second imaging was 6 h 46 min, mean time from symptom onset to third imaging was slightly more than 2 days, and the mean time from symptom onset to the final MRI was 26 days (see Table 1 for clinical data).

**Table 1:** Patient characteristics.

**Patient, *n***	**21**
Gender female, *n* (%)	10 (48)
Age, MV ± SD [years]	65 ± 14
T1 [hh:mm], MV ± SD [hh:mm]; range [hh:mm]	03:09 ± 01:38; 01:11–06:43
T2 [hh:mm], MV ± SD [hh:mm]; range [dd:hh:mm]	06:46 ± 04:31; 00:04:08–01:01:29
T3 [dd:hh:mm], MV ± SD [dd:hh:mm]; range [dd:hh:mm]	02:03:47 ± 01:01:38; 01:03:13–05:00:36
T4 [dd:hh:mm], MV ± SD [dd:hh:mm]; range [dd:hh:mm]	26:04:00 ± 20:21:36; 07:01:45–31:23:59
Side of infarction, left *n* (%)	10 (48)
Lesion volume	
Acute DWI lesion—T1 [ml], MW ± SD; range (ml)	25.8 ± 36.8; 1.34–134.79
Final infarct—T3 [ml], MW ± SD; range (ml)	45.2 ± 69.2; 1.15–256.88
Reversible diffusion lesion [ml], Median (IQR)	2.9 (1.65–6.98)
Persistent core [ml], Median (IQR)	5.3 (0.93–15.32)
Infarct growth [ml], Median (IQR)	4.6 (3.50–22.09)
iv-thrombolysis, *n* (%)	10 (48)
NIHSS (T1), median [IQR]	7 [4–12]

*Values are: MV, mean value± SD, standard deviation or median and interquartile range [IQR] and range; T1, time point 1, from symptom onset to first imaging on admission (1–7 h post-onset); T2, time point 2, from symptom onset to second imaging (4–26 h post-onset); T3, time point 3, from symptom onset to third imaging (1–5 days post-onset); T4, time point 4, from symptom onset to fourth imaging (7–33 days post-onset)*.

**Figure 3:**
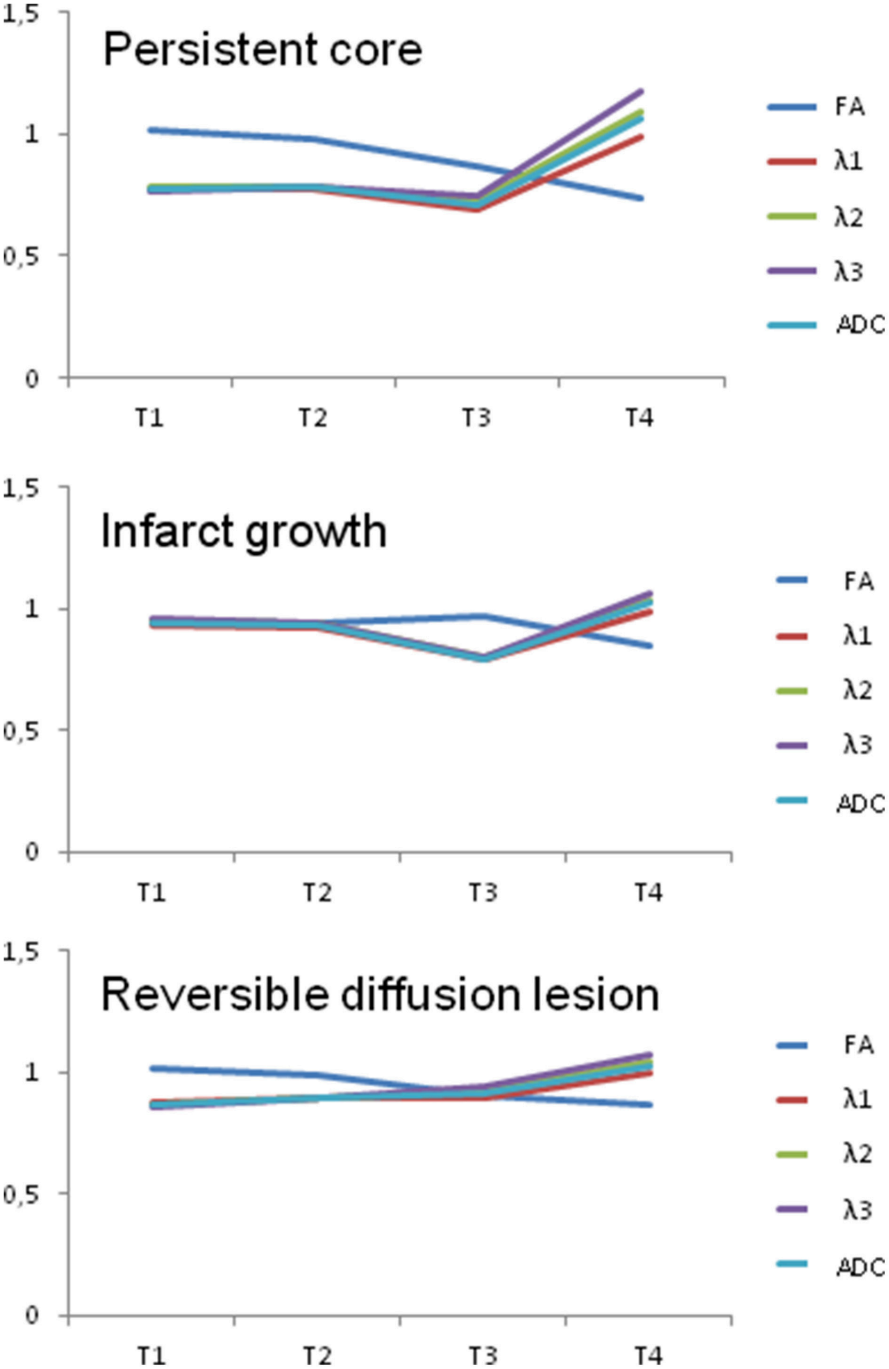
This figure shows the ratio of affected and unaffected side in the different infarct lesions (core, growth, and reversible lesion) of FA, ADC, and eigenvalues (λ1, λ2, and λ3) at timepoint 1–4 (with T1: time point 1, from symptom onset to first imaging on admission (1–7 h post-onset); T2: time point 2, from symptom onset to second imaging (4–26 h post-onset); T3: time point 3, from symptom onset to third imaging (1–5 days post-onset); T4: time point 4, from symptom onset to fourth imaging (7–33 days post-onset).

In the persistent core, FA ratio (rFA) was slightly increased at T1 and continuously decreased until T4, whereas ratios of ADC (rADC) and eigenvalues (rλ1, rλ2, rλ3) were markedly decreased already at T1, showed some further decrease at T3, and finally increased to near normal or above 1 at T4 (see Figure 3). There was a significant difference of rFA in the persistent core between the different time points [F_(2.16, 40.9)_ = 26.05, *p* < 0.0005].

In the area of infarct growth, rFA was slightly decreased at T1 to T3 with a further decrease toward T4. Ratios of ADC and eigenvalues showed a slight decrease at T1 and T2 followed by a stronger decrease at T3, and returned to values close to or above 1 at T4. rFA in the area of infarct growth differed statistically significantly between the different time points [F_(3, 57)_ = 8.44, *p* < 0.0005].

In the brain area with reversible diffusion lesion, rFA ratio was minimally increased at T1 and showed a continuous decreased toward T4. In contrast, rλ1, rλ2, rλ3 were decreased at T1 and exhibited a continuous increase at T2 and T3 reaching values close to or above 1 at T4 (see Table 2). In Table 2, the ratios of diffusion parameter are presented for the different tissue compartments and time-points. As for the other two compartments, rFA in the reversible lesion was significantly different between the different time points [F_(3, 57)_ = 9,889, *p* < 0.0005). Pairwise comparison using Bonferroni correction for multiple comparisons revealed that rFA in the reversible lesion at T4 was significantly smaller than rFA at T1 (*p* = 0.003) and T2 (*p* = 0.012). rFA at T3 was also significantly smaller than rFA at T1 (*p* = 0.028).

**Table 2:** Ratio of affected and unaffected side of FA, ADC, and eigenvalues (λ1, λ2, and λ3) in the different tissue compartments (core, growth, and reversible lesion) over time.

		**rFA**	**rλ1**	**rλ2**	**rλ3**	**rADC**
Core	T1	1.015 ± 0.152	**0.772 ± 0.085**	**0.781 ± 0.093**	**0.764 ± 0.097**	**0.772 ± 0.088**
	T2	0.983 ± 0.123	**0.776 ± 0.110**	**0.788 ± 0.120**	**0.786 ± 0.125**	**0.781 ± 0.116**
	T3	0.871 ± 0.184	**0.688 ± 0.120**	**0.725 ± 0.119**	**0.750 ± 0.139**	**0.713 ± 0.122**
	T4	**0.740 ± 0.202**	0.991 ± 0.115	1.094 ± 0.150	1.173 ± 0.195	1.067 ± 0.134
Growth	T1	0.954 ± 0.094	**0.933 ± 0.044**	**0.952 ± 0.052**	0.956 ± 0.064	**0.944 ± 0.046**
	T2	0.939 ± 0.103	**0.919 ± 0.064**	0.940 ± 0.074	0.939 ± 0.078	**0.931 ± 0.068**
	T3	0.965 ± 0.144	**0.789 ± 0.098**	**0.804 ± 0.091**	**0.798 ± 0.100**	**0.795 ± 0.093**
	T4	**0.850 ± 0.129**	0.987 ± 0.071	1.034 ± 0.072	1.066 ± 0.088	1.022 ± 0.070
Rev	T1	1.016 ± 0.143	**0.876 ± 0.072**	**0.869 ± 0.072**	**0.861 ± 0.084**	**0.871 ± 0.070**
	T2	0.984 ± 0.092	**0.895 ± 0.093**	**0.895 ± 0.095**	**0.897 ± 0.094**	**0.896 ± 0.092**
	T3	0.900 ± 0.151	**0.896 ± 0.090**	0.926 ± 0.105	0.943 ± 0.136	0.917 ± 0.099
	T4	0.870 ± 0.185^*^	0.996 ± 0.055	1.043 ± 0.101	1.071 ± 0.133	1.030 ± 0.082

All values are ratios between affected and unaffected side given as mean value ± standard deviation; values highlighted in bold were significantly different between the affected side and the unaffected side in exploratory analysis using t-test for dependent samples with p < 0.001;

* *significantly different between the affected side and the unaffected side using t-test for dependent samples p = 0.001; T1, time point 1, from symptom onset to first imaging on admission (1–7 h post-onset); T2, time point 2, from symptom onset to second imaging (4–26 h post-onset); T3, time point 3, from symptom onset to third imaging (1–5 days post-onset); T4, time point 4, from symptom onset to fourth imaging (7–33 days post-onset), fourth imaging; core, persistent core; growth, Infarct growth; rev, reversible diffusion lesion; ADC, apparent diffusion coefficient; FA, fractional anisotropy*.

The mixed random intercept model identified a significant influence of the tissue compartment on the ratio values of all diffusion parameters except for rFA. Time-point had a significant influence on all parameters (i.e., ratio of FA, ADC and eigenvalues). Finally, a significant interaction of time-point and tissue compartment was observed for all parameters (see Table 3).

**Table 3:** Analysis of influence of mask and time point on the values (ratio of affected and unaffected side) of FA, ADC, and eigenvalues (λ1, λ2, and λ3).

	**FA**	**λ1**	**λ2**	**λ3**	**ADC**
Mask (cor, rev, gro), *p*	0.34	<0.001	<0.001	0.001	<0.001
Time point, *p*	<0.001	<0.001	<0.001	<0.001	<0.001
Interaction between mask and time point, *p*	0.009	<0.001	<0.001	<0.001	<0.001

*cor, persistent core (consistent infarct area at time point 1 and 3); rev, reversible diffusion lesion (infarct area in remission between time point 1 and 3); gro, Infarct growth (growth of infarct area between time point 1 and 3); ADC, apparent diffusion coefficient; FA, fractional anisotropy*.

## Discussion

We analyzed dynamic changes of diffusion parameters in acute ischemic stroke patients from first hours of stroke onset up to a chronic state after 1 month in different lesion compartments. As a main result, specific time courses of FA, ADC, and diffusion eigenvalues were observed in the persistent core, the area of infarct growth and the region of reversible diffusion lesion volume, which differed both between diffusion indices and tissue compartments.

As expected, in the persistent core rADC and the ratios of the eigenvalues were reduced already within the first hours, returning to normal or even elevated levels 1 month after stroke. In contrast, a decrease of rFA was observed with a certain delay and becoming more pronounced as time evolved. This is in line with pathophysiological models of water diffusion in cerebral ischemia and previous reports (15). ADC, as a measure of the freedom of water diffusion, is reduced in cerebral ischemia because of a shift of water, from the extracellular to the intracellular compartment in acute stroke (15). With progressive damage to tissue structure and consecutive destruction of organized diffusion barriers occurring later on, FA as a measure of directionality of diffusion decreases with a certain delay. During the further course with elimination of cell debris, finally several days to weeks after stroke, ADC and eigenvalues are increased in the now necrotic tissue, while FA remains decreased (16). This course of water diffusion changes following stroke has already described both in experimental stroke (17) as well as in human stroke (18).

Within the area of infarct growth we observed a similar but delayed direction of diffusion changes as in the infarct core. In the acute and very early follow-up MRI within the first hours of stroke, only a minor decrease of rADC and ratio of eigenvalues was observed, which became pronounced during the follow-up after 2 days. This is in line with the observation that the tissue of these brain areas only turned into infarction after 2 days, consistent with the interpretation of infarct growth into the area of penumbra (19). However, it appears noteworthy that although the brain tissue in this area was considered normal and not affected by the persistent core based on visual assessment, a slight decrease of rADC and ratio of eigenvalues could already be demonstrated in quantitative analysis. This observation might be explained by a subtle alteration of diffusion parameters in a brain area of critical hypoperfusion which remained above the threshold of structural tissue damage and of visual detection during the first hours of stroke (20).

The third tissue compartment studied was defined by a reversible diffusion lesion, i.e., the part of the initial diffusion lesion that is considered to be part of the penumbra and not of the persistent core (21). Partial DWI normalization is a well described phenomenon which may occur in up to 20% of stroke patients (22), while the clinical relevance of this observation is unclear (23). In our analysis, the reversed lesion compartment exhibited reduced rADC and ratio of eigenvalues in the acute MRI examination within the first hours consistent with its involvement in the acute diffusion lesion. However, in contrast to the development of diffusion changes in the other compartments, an increase of rADC and ratio of eigenvalues was already observed within the first days of ischemia, reflecting the visual observation of a “normalization” of the diffusion lesion. Nevertheless, diffusion indices remained reduced, even if this slight reduction escaped visual observation. Continuously decreasing FA which is used as a marker of truly irreversible tissue damage confirms the interpretation that the brain tissue in this compartment does not show full reconstitution but exhibits at least signs of partial tissue damage. This is in line with serial studies of brain areas with DWI normalization that demonstrated MRI characteristics of ischemic injury in repeated imaging after 7 days interpreted as “late ischemic injury” (24). Our findings together the previous reports of ischemic injury in tissue with reversible DWI lesion (25) point toward partial or delayed damage to these brain areas that may be located in the border zone of the infarct core. These findings may reflect selective neuronal loss as previously revealed by Imonazenil-SPECT in brain regions with perfusion-diffusion mismatch escaping infarction (26).

Comparing the changes between the different diffusion indices, there was a significant effect of time for all parameters (ratio of FA, ADC and eigenvalues) while the tissue compartment was only significant for rADC and ratio of eigenvalues, but not for rFA. This may result from the fact that FA might be less sensitive to very early effects of ischemia on water diffusion in brain tissue prior to the occurrence of irreversible damage to tissue structures. While ADC changes in acute cerebral ischemia are well understood and monodirectional, both increased and decreased FA has been reported in acute ischemic stroke (27–30). Only after irreversible tissue damage has occurred, FA demonstrates a clear decrease with no normalization over time. Thus, within the very first hours of ischemia FA does not help in defining tissue status, but as soon as FA shows a marked decrease (which is usually beyond 12 h) it can be considered as an indicator of truly irreversible tissue damage (31).

The results of our analysis have to be interpreted with caution, as there are numerous factors influencing tissue fate in acute cerebral ischemia which we do not capture in our study. We did not take into account lesion location or the involvement of gray or white matter, which may influence the time course of diffusion parameters in cerebral ischemia (32, 33), nor did we have information on tissue perfusion available for this analysis. Other diffusion models as for example NODDI, which we did not use, are able to add value to standard DTI, for example by revealing specific microstructural substrates to white matter changes detected with FA (34). Further dependency of the absolute ADC and relative DWI thresholds used on absolute lesion volumes and changes in lesion volumes over 24 h after recanalization is described (35). Although we have chosen day 2 to define the final infarct region, we cannot exclude that atrophy occurring at time point 4 influences DTI results. As CSF was excluded based on the DWI sequence acquired with a higher slice thickness than the DTI sequence, we cannot fully exclude a potential contamination by CSF signal. Finally, the small sample size makes our study susceptible to bias.

## Conclusion

Diffusion tensor imaging displays characteristic changes of water diffusion in different tissue compartments in acute ischemic stroke. Also regions with reversible diffusion lesion show diffusion signatures of persisting tissue alterations.

## Author Contributions

AA and GT: make substantial contributions to conception and design and acquisition of data and analysis and interpretation of data, participate in drafting the article and give final approval of the version to be submitted and any revised version; SM: make substantial contributions to acquisition of data and analysis and interpretation of data, participate in drafting the article and give final approval of the version to be submitted and any revised version; HB: make substantial contributions to conception and analysis and interpretation of data, participate in drafting the article and give final approval of the version to be submitted and any revised version; SG, T-HC, LD, and YB: make substantial contributions to acquisition of data, participate in drafting the article and give final approval of the version to be submitted and any revised version; MH: make substantial contributions acquisition of data, participate in drafting the article and give final approval of the version to be submitted and any revised version; NN: make substantial contributions to conception and design and acquisition of data, participate in drafting the article and give final approval of the version to be submitted and any revised version; CG: make substantial contributions to conception and design, participate in drafting the article and give final approval of the version to be submitted and any revised version; JF: make substantial contributions to conception and design and acquisition of data and interpretation of data, participate in drafting the article and give final approval of the version to be submitted and any revised version.

### Conflict of Interest Statement

The authors declare that the research was conducted in the absence of any commercial or financial relationships that could be construed as a potential conflict of interest.
